# Personal

**Published:** 1889-03

**Authors:** 


### Personal.

Dr. John Parmenter, of Buffalo, who has been studying abroad for the past two years, arrived home in the latter part of February. During his absence, Dr. Parmenter has been the pupil of Billroth and Albert in Vienna, Max Schede in Hamburg, Thiersch in Leipsic, and Keitly, Morris and Lister in London. It will be seen that he has paid particular attention to surgery, and we understand will devote himself hereafter exclusively to that work.

---

Dr. James W. Putnam, of this city, has returned to his home after two years spent in Europe pursuing the special study of diseases of the nervous system. Coming fresh from the teachings of Nothnagel, Rosenthal, Westphal, Mendel, Charcot,

Brown-Sequard, Hughlings-Jackson, Gowers, Ferrier, Horsely, and others, he returns with ample equipment for the practice of his chosen specialty, to which he will devote his time in the future.

---

DR. CHARLES A. L. REED, of Cincinnati; Dr. Herman Biggs, ot New York; and Dr. Herman Mynter, of this city, will read papers on professional subjects before the Alumni Association ot Niagara University, Medical Department, April 9, 1889.

